# Antioxidant defense network and toxicity risk assessment in *Panax* species under heavy metals stress

**DOI:** 10.3389/fpls.2026.1860085

**Published:** 2026-06-15

**Authors:** Zhongzhou Yang, Kedong Xu, Changjun Deng, Xiaoyan Li, Honghong Gao

**Affiliations:** 1 Key Laboratory of Crop Molecular Breeding and Bioreactor, Zhoukou Normal University, Zhoukou, China; 2 Yunnan Hanzhe Technology Co., Ltd, Kunming, China; 3 Department of Clinical Laboratory, Heping Branch, Shanxi Provincial People’s Hospital, Taiyuan, China; 4 Inpatient Service Center, Shanxi Provincial People’s Hospital, Taiyuan, Shanxi, China

**Keywords:** Chinese medicinal materials, machine learning, medicinal plants, metal pollution, toxicology

## Abstract

The continuous accumulation of heavy metals (HMs) in soil poses a serious threat to the quality and safety of medicinal plants. However, a systematic assessment of the responses of the antioxidant defense system in *Panax* medicinal plants under HM stress is still lacking. In this study, we integrated 641 pairs of control-observation data from global sources and systematically analyzed the response characteristics of *Panax* medicinal plants under HM stress using meta-analysis and machine learning modeling. The results showed that HM exposure significantly promoted catalase activity (11.63%, *P* < 0.05), gene expression levels (56.10%, *P* < 0.01), and heavy metal accumulation (218.55%, *P* < 0.001) in *Panax* medicinal plants, ultimately leading to irreversible biomass loss (−29.62%, *P* < 0.001). The full RF model exhibited good fit (R^2^ = 0.66, RMSE = 0.44), while a reduced model with only environmental predictors showed low predictive performance (R^2^ = 0.18). Therefore, the analysis is more appropriate for identifying the relative importance of modulating factors than for robust toxicity prediction. SHAP analysis and PLS-PM revealed that HM concentration (12.33%), geographical characteristics (11.93%), and duration of metal pollution exposure (5.53%) are key factors regulating the antioxidant defense system of *Panax* medicinal plants. This study elucidates the response mechanisms of the antioxidant defense system in *Panax* medicinal plants under HM stress, providing a scientific basis for toxicity risk assessment, resistant variety screening, and the development of field regulation strategies.

## 1 Introduction

With the rapid development of industrialization and agricultural intensification, the input of heavy metals (HMs, e.g., cadmium: Cd, lead: Pb, arsenic: As, copper: Cu, etc.) into the soil environment has been continuously increasing (Hu et al., 2013; Wang et al., 2020; Ullah et al., 2023), making it one of the key environmental issues threatening ecological security and sustainable agricultural development worldwide (Luo et al., 2026; Wu et al., 2025). According to statistics from the FAO, millions of hectares of farmland globally are contaminated with HMs to varying degrees, with particularly prominent heavy metal exceedances in soils around mining areas and suburban farmlands, especially in developing countries (Roy et al., 2025; Xu et al., 2017). HMs are characterized by concealment, persistence, and bioaccumulation (Tarfeen et al., 2022); they not only threaten human health through the food chain but also exert significant toxic effects on plant growth and development (Cheng et al., 2025; Li et al., 2014; Wang et al., 2025a). Therefore, systematically assessing the toxicity mechanisms of HMs on important economic plants has become a research hotspot in the fields of environmental science and plant protection.

The *Panax* genus of medicinal plants includes *P. notoginseng*, *P. quinquefolius*, *P. japonicus*, and *P. vietnamensis*, which are important sources of valuable traditional Chinese medicinal materials (Jee et al., 2014; Chan et al., 2019; Yang et al., 2018; Xia et al., 2016). Due to their stringent growth requirements and long rotation cycles, the main production areas of *Panax* plants often face the risk of soil heavy metal accumulation (Geng et al., 2023; Huang et al., 2023b). Studies have shown that exposure to HMs can lead to blocked photosynthesis (Liu et al., 2025), reduced biomass (Zu et al., 2022), reactive oxygen species (ROS) burst, and membrane lipid peroxidation in *Panax* plants (Lu et al., 2023; Huo et al., 2021), and in severe cases, even plant death (Wang et al., 2026). To cope with oxidative stress, plants activate their antioxidant defense system, including the synergistic action of antioxidant enzymes such as superoxide dismutase, peroxidase, and catalase (Ali et al., 2006; Tewari et al., 2008), as well as the transcriptional regulation of related genes (Balusamy et al., 2013). However, current research on the antioxidant defense responses of *Panax* under HM stress mostly focuses on a single metal, a single indicator, or a single species, lacking a cross-study systematic integration of multiple metals, multiple indicators, and multiple species, making it difficult to draw universal conclusions.

Traditional review studies, although capable of qualitatively summarizing relevant findings, cannot quantify the overall effect size and uncertainty of HM stress (Zhang et al., 2023; Tian et al., 2024). In recent years, meta-analysis has been widely applied in the field of ecotoxicology, enabling effective integration of multi-source independent study data, quantification of comprehensive effect sizes, and identification of heterogeneity sources (Wang et al., 2024; Huang et al., 2023a; An et al., 2023). Furthermore, machine learning methods such as random forest (RF) have unique advantages in handling high-dimensional nonlinear relationships, allowing further revelation of key drivers affecting toxicity effects (Tasan et al., 2024; Zhu et al., 2025). Partial least squares path modeling (PLS-PM) can resolve the direct and indirect pathways among multiple variables, providing methodological support for elucidating complex regulatory networks (Zhang et al., 2024; Sun et al., 2023). By systematically integrating multiple analytical methods, this study aims to overcome the sample size limitations and conclusion biases of individual studies, thereby enabling a comprehensive toxicity risk assessment and key factor analysis.

Based on this, this study systematically integrated relevant literature and experimental data from a global scale, comprehensively evaluated the responses of the antioxidant defense system of *Panax* medicinal plants under HM stress using meta-analysis, and constructed a machine learning toxicity prediction framework. The specific research objectives include: (1) systematically quantifying the effects of HM stress on the physiological functions, antioxidant enzyme activities, and gene expression of *Panax* medicinal plants; (2) identifying key environmental variables affecting the toxicity intensity in *Panax* medicinal plants and establishing a machine learning framework capable of predicting toxicity effects; (3) elucidating the effects of sensitive environmental factors on key antioxidant enzymes and their hierarchical regulatory networks.

## 2 Materials and methods

### 2.1 Data retrieval

To investigate the toxic responses of *Panax* medicinal plants under metal stress, we selected the Web of Science, PubMed, and Google Scholar databases to collect literature from their inception to March 20, 2026. The search keywords were as follows: (“Panax” OR “*Panax notoginseng*” OR “*Panax quinquefolius*” OR “*Panax japonicus*” OR “*Panax vietnamensis*”) AND (“Metal” OR “Cd” OR “Pb” OR “As” OR “Hg” OR “Cr” OR “Cu” OR “Zn” OR “Ni”) AND (“toxicity” OR “stress” OR “accumulation” OR “gene expression”). The network visualization of the literature retrieval results is shown in **Supplementary Figure 1**. The literature inclusion criteria were as follows: (1) the research subjects were *Panax* plants; (2) the experimental group was exposed to HMs, while the control group was under the same culture conditions without metal addition; (3) at least one toxicity endpoint related to plant antioxidant defense or gene expression was reported, and the data were presented as mean, standard deviation (or standard error), and sample size; (4) reviews, conference abstracts, dissertations, and non-Chinese/English literature were excluded. The complete screening process is shown in **Supplementary Figure 2**. Through the above screening, a total of 641 pairs of control-observation data were extracted.

### 2.2 Meta analysis

The natural log-transformed response ratio (ln*R*) was used as the effect size to quantify the impact of HM exposure on various toxicity endpoints in *Panax* plants (An et al., 2024). The effect size (ln*R*) and its variance (Var ln*R*) were calculated using **Equations 1**, **2** (Che et al., 2024):

(1) $$\ln R=\ln\left({\bar{X}}_{T}/{\bar{X}}_{C}\right)$$

(2) $$\mathrm{Var}\left(\ln R\right)=\frac{SD_{T}^{2}}{n_{T}{\bar{X}}_{T}^{2}}+\frac{SD_{C}^{2}}{n_{C}{\bar{X}}_{C}^{2}}$$

Where $\bar{X}$ are the mean values; n is the sample size; SD is the standard deviation.

If the data reported in the included literature were presented as standard error, they were first converted to standard deviation for subsequent analysis. The calculation method is shown in **Equation 3** (Lv et al., 2025):

(3) $$\text{SD}=\text{SE}\times \sqrt{\text{n}}\;$$

To more intuitively interpret the magnitude of the impact of HM exposure on *Panax* medicinal plants, the effect size was converted into percentage change using **Equation 4** for reporting (Zhen et al., 2025):

(4) $$\text{RR}\%\;=\;({\text{e}}^{\ln R}-1)\times 100\%$$

The full set of toxicity endpoints included in the meta-analysis encompassed 22 parameters grouped into four categories: (1) Physiological stress: biomass, plant height, root length, survival rate, net photosynthetic rate (Pn), transpiration rate (Tr); (2) Antioxidant enzyme: superoxide dismutase (SOD), peroxidase (POD), catalase (CAT), ascorbate peroxidase (APX), glutathione reductase (GR); (3) Gene expression: SOD gene (SOD1 and SOD2), *Panax* glutathione peroxidase gene (PgGPX), *Panax* APX gene (PgAPX), *Panax* CAT gene (PgCAT), cytochrome P450 transcription level (P450 transcription); (4) Stress and Metabolism: malondialdehyde (MDA), hydrogen peroxide (H_2_O_2_), reduced glutathione (GSH), adenosine triphosphate (ATP), metal accumulation.

Based on the Akaike Information Criterion (AIC), a random-effects model was selected for data synthesis. Since the effect sizes were not normally distributed (Kolmogorov-Smirnov test, *P* < 0.001), a weighted bootstrap resampling method (number of iterations = 999) was used to calculate the 95% confidence intervals (CI) of the effect sizes (Huang et al., 2023a). If the CI included 0, the effect was considered not statistically significant. The rationale for subgroup partitioning was assessed using the between-group heterogeneity test (Q_b_< 0.001), and significant heterogeneity between subgroups was considered when the Q_b_ test had a *P* < 0.05. Publication bias was evaluated using Rosenthal’s fail-safe coefficient (An et al., 2023). The fail-safe coefficient was 41,075.5 (far greater than the critical value of 5n + 10 = 3,215), indicating no significant publication bias. To quantify the heterogeneity among studies, standard statistics were computed. The Q statistic was 212,617.5 (df = 640, *P* < 0.001), indicating highly significant heterogeneity across all effect sizes. The estimated between-study variance was τ^2^ = 0.77, and the proportion of total variation attributable to true heterogeneity was I^2^ = 99.84%. This very high I² reflects the substantial diversity of experimental conditions, plant species, HM types and concentrations, and toxicity endpoints integrated in this study, and further justifies the use of a random-effects model and the subsequent subgroup analyses to explore sources of heterogeneity.

Given that multiple effect sizes were often extracted from the same study, we performed a sensitivity analysis by randomly selecting one effect size per study (1,000 iterations) and recomputing the pooled effect sizes. Across all iterations, the sign (positive/negative) of the pooled effect sizes remained consistent with the original analysis in > 98% of iterations, and the median deviation from the original effect sizes was< 15% for all endpoints (maximum< 30%). These results suggest that potential pseudoreplication does not substantially bias our primary conclusions. We acknowledge that multilevel meta-analysis or robust variance estimation would be more rigorous, but software constraints (MetaWin 2.1) and the unbalanced distribution of effect sizes per study (median = 3, range 1–24) precluded their application. Therefore, we retained the conventional random-effects model and discuss this limitation in Section 3.4.

### 2.3 Machine learning

To construct a predictive model for the toxic effects of HMs on *Panax* plants, this study employed the RF algorithm. RF, as an ensemble learning method, effectively handles high-dimensional features and nonlinear relationships by constructing multiple decision trees and synthesizing their predictions, and it exhibits strong robustness against overfitting (Dang et al., 2022; Chen et al., 2024; Shaik et al., 2022). The detailed workflow of model construction is shown in **Supplementary Figure 3**.

Among the included studies, over 80% were pot or laboratory experiments, with only a small proportion conducted under field conditions. Despite this, geographic coordinates were retained as predictor variables because, even in controlled experiments, the soil substrate is typically collected from specific geographic locations and thus carries the inherent physicochemical properties, background HM levels, and microbial profiles of the region of origin. Geographic coordinates therefore serve as integrative proxies for regional environmental factors that may influence plant basal physiological status and metal bioavailability. In model construction, environmental variables affecting the antioxidant defense system were used as predictors input into the model, including nine variables: metal type (categorical), metal concentration (continuous, mg kg^-1^), exposure duration (continuous, days), exposed object (categorical, *Panax* species), field management (categorical, encoding specific amelioration practices reported in the source studies: none/control, calcium application, biochar amendment, potassium fertilization, phosphorus fertilization, and others), longitude and latitude (continuous, representing the experimental site for field studies or the soil collection site for pot studies, serving as proxies for regional background soil properties and climate), and response indicator (categorical). All categorical variables were one-hot encoded, and continuous variables were standardized (z-score) prior to model training. No missing data were present for these predictor variables. The dataset was divided into a training set (80%) and a testing set (20%) using stratified random sampling to ensure that different plant species and heavy metal types were consistently distributed across both groups. Grid search combined with ten-fold cross-validation was employed for hyperparameter optimization of the RF model. Ten-fold cross-validation randomly partitioned the training set into 10 equal-sized subsets. In each iteration, 9 subsets were used to train the model and the remaining 1 subset was used to validate performance. This process was repeated 10 times, and the average was taken as the performance evaluation metric for each hyperparameter combination. This approach ensured the stability of model prediction. Finally, the coefficient of determination (R^2^) value and root mean square error (RMSE) served as the criteria for evaluating model accuracy. Calculate using **Equations 5**, **6**.

(5) $${\text{R}}^{2}=\frac{{\left[\sum_{i=1}^{n}\left(x_{\left(i\right)}-x_{m}\right)\left(y_{\left(i\right)}-y_{m}\right)\right]}^{2}}{\sum_{i=1}^{n}{\left(x_{\left(i\right)}-x_{m}\right)}^{2}\sum_{i=1}^{n}{\left(y_{\left(i\right)}-y_{m}\right)}^{2}}$$

(6) $$\text{RMSE}=\sqrt{\frac{\sum_{\text{i}=1}^{\text{n}}{\left({\text{pe}}_{\text{i}}-{\text{p}}_{\text{i}}\right)}^{2}}{\text{n}}}$$

To evaluate the contribution of predefined endpoint categories to model performance, a reduced RF model was also constructed excluding the categorical variables response indicator and category group, retaining only environmental and experimental predictors (metal type, metal concentration, exposure duration, exposed object, field management, longitude, and latitude). Meanwhile, the SHapley Additive exPlanations (SHAP) method was used to interpret model predictions and quantify the contribution of each input feature to the effect size (Zhang et al., 2025b). SHAP values, rooted in game theory’s Shapley value, attribute an influence score to each feature per prediction. Positive contributions raise the predicted outcome, while negative contributions lower it (Chi et al., 2023; Zhuang et al., 2025). The mean absolute SHAP value of each feature across all samples was calculated as the global feature importance.

PLS-PM was used to analyze the overall responses of *Panax* medicinal plants to four latent variables: geographical characteristics, field management, metal characteristics, and exposure level. Geographical characteristics was a reflective construct formed by the longitude and latitude of the experimental site (field studies) or soil collection site (pot studies), serving as a proxy for regional environmental factors such as pollution background, climate, and soil properties. Field management was a single-indicator construct based on the categorical variable encoding specific amelioration practices (none/control, calcium application, biochar amendment, potassium fertilization, phosphorus fertilization, and others). Metal characteristics was a formative construct comprising metal type (categorical) and metal concentration (continuous, mg kg^-1^). Exposure level was a formative construct comprising exposure duration (continuous, days) and exposed object (categorical, *Panax* species). All path coefficients represent associations consistent with the hypothesized structural model and should not be interpreted as confirmed causal effects.

### 2.4 Statistical analysis

IBM SPSS Statistics 27 was used to perform normality testing of the data (Kolmogorov-Smirnov test). Meta-analysis was completed using MetaWin 2.1 software. The RF model was constructed based on Python 3.12 (scikit-learn 1.6.0 library), and SHAP analysis was performed using the shap 0.46.0 library. All statistical tests were two-tailed. Data visualization was completed using R 4.3.3 (ggplot2 3.5.0 package) and Python Matplotlib 3.8.0.

## 3 Results and discussion

### 3.1 Overall effects of HM exposure on *Panax* medicinal plants

**Figure 1:**
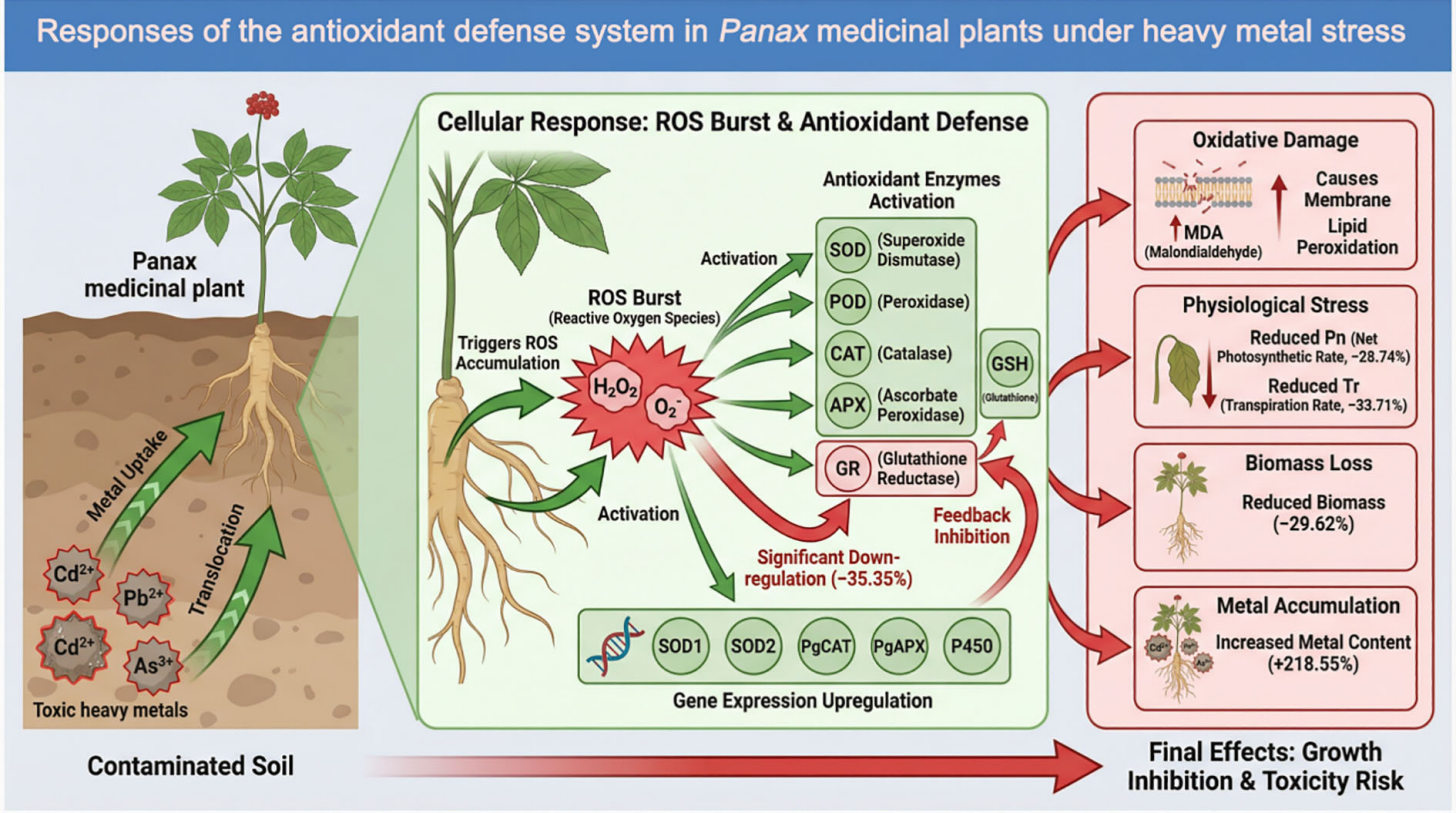
Schematic diagram of HM-induced responses of the antioxidant defense system in *Panax* medicinal plants.

**Figure 2:**
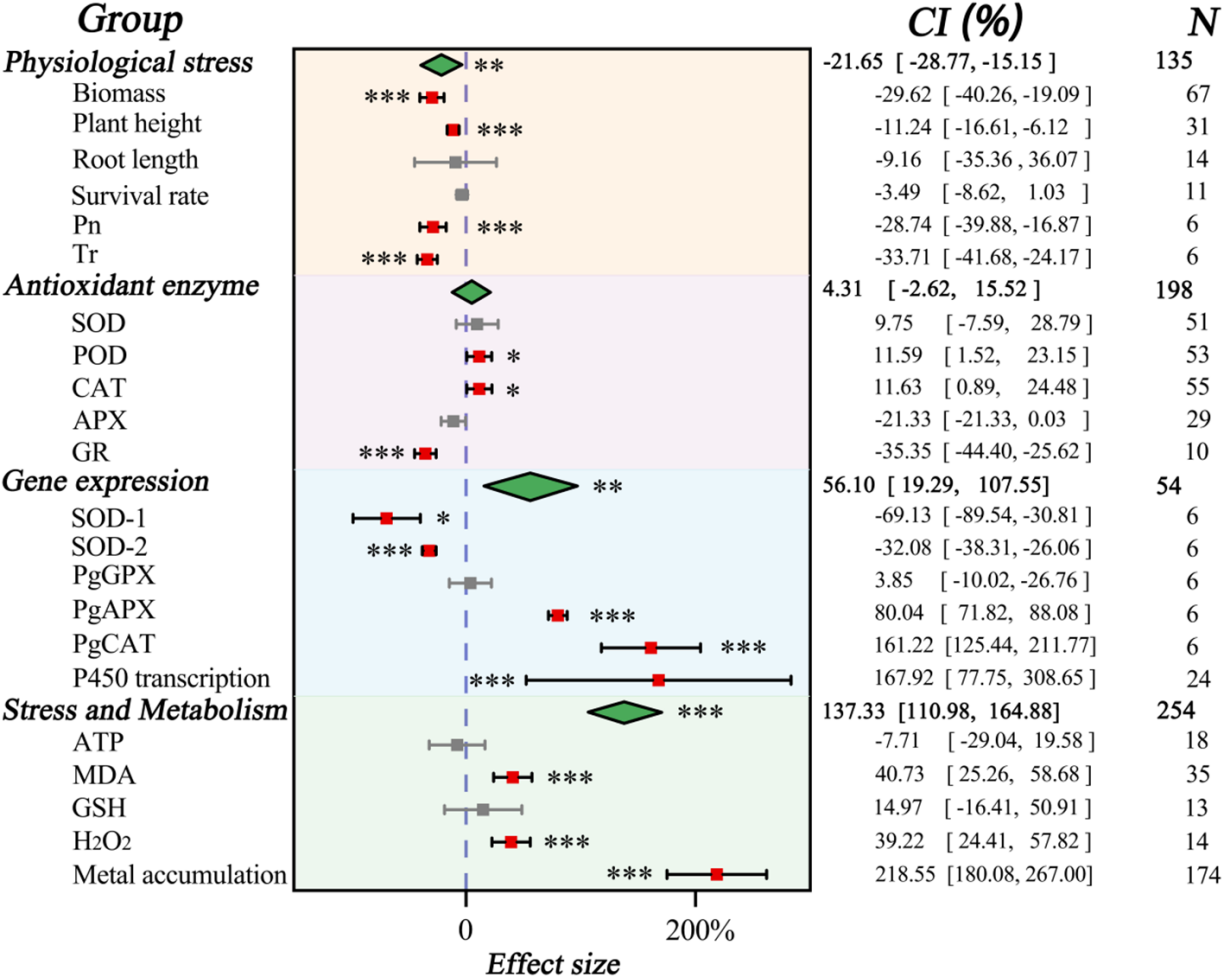
Meta-analysis of the ecotoxicity of HMs on *Panax* medicinal plants. Effect sizes represent the percentage changes in toxicity indicators of *Panax* medicinal plants under HM exposure. Error bars represent 95% bootstrap confidence intervals (999 iterations). Pn, net photosynthetic rate; Tr, transpiration rate; SOD, superoxide dismutase; POD, peroxidase; CAT, catalase; APX, ascorbate peroxidase; GR, glutathione reductase; SOD1 and SOD2, SOD gene; PgGPX, *Panax* glutathione peroxidase gene; PgAPX, *Panax* APX gene; PgCAT, *Panax* CAT gene; P450, cytochrome P450 transcription level; ATP, adenosine triphosphate; MDA, malondialdehyde; GSH, reduced glutathione; H_2_O_2_, hydrogen peroxide.

**Figure 1** comprehensively illustrates the process of the toxic response of *Panax* medicinal plants to HM pollution in the soil environment. To systematically evaluate the toxic effects of HM exposure on *Panax* medicinal plants and their antioxidant defense response mechanisms, this study employed a meta-analysis approach, integrating 641 pairs of control-observation data covering four major categories (Physiological stress, Antioxidant enzyme, Gene expression, Stress and Metabolism) with a total of 22 toxicity endpoints. **Figure 2** presents the percentage change (RR%) and 95% confidence intervals for each toxicity indicator under HM exposure. Overall, the responses of different indicators to HM stress exhibited significant heterogeneity, reflecting the complex and multi-layered regulatory network of *Panax* plants at the physiological, biochemical, and molecular levels. It should be noted that the overall heterogeneity of this meta-analysis is extremely high (I^2^ = 99.84%), reflecting substantial differences across the included studies in terms of toxicity endpoint types, plant species, heavy metal types and concentrations, exposure conditions, and other factors. Therefore, the pooled effect sizes for each overall category presented above should be interpreted as average tendencies rather than overgeneralized as single conclusions. In practice, priority should be given to the subgroup effect sizes for specific toxicity indicators along with their confidence intervals (as shown in **Figure 2**), and comprehensive interpretation should integrate the results of subsequent subgroup analyses and machine learning.

#### 3.1.1 Exposure to HMs exacerbates plant physiological stress

The effects of HM exposure on indicators within the physiological stress group of *Panax* medicinal plants are shown in **Figure 2**. The combined effect across this group showed a significant inhibition of −21.65% (*P* < 0.01), indicating that HM ions (e.g., Cd^2+^, Pb^2+^, As^3+^) significantly impair the growth performance of *Panax* plants by interfering with the photosynthetic system, water metabolism, and biomass accumulation. Among the specific indicators, Pn and Tr decreased by 28.74% (*P* < 0.001) and 33.71% (*P* < 0.001), respectively, representing the two indicators with the largest response magnitudes within the physiological stress group. The significant inhibition of photosynthesis may be related to HM-induced stomatal closure, chlorophyll degradation, and damage to the photosystem II reaction center (Papazoglou et al., 2005; Gan et al., 2023). The decrease in transpiration rate further limits the absorption of water and mineral nutrients, creating a vicious cycle (Schuck and Greger, 2020; Tang et al., 2023). Biomass also showed a significant decrease (−29.62%, *P* < 0.001), which is directly associated with the inhibition of photosynthesis. In contrast, the decrease in plant height was smaller (−11.24%, *P* < 0.001), suggesting that *Panax* plants may prioritize maintaining longitudinal growth at the expense of biomass accumulation under metal stress. Although root length (−9.16%) and survival rate (−3.49%) showed decreasing trends, neither reached statistical significance (*P* > 0.05). This may be related to their relatively small sample sizes (root length: *n* = 14; survival rate: *n* = 11) or the stronger tolerance of *Panax* plants regarding root growth and basic survival ability under short-term exposure. Overall, HM exposure impairs the growth performance of *Panax* medicinal plants primarily through the inhibition of photosynthesis (Pn and Tr) and biomass accumulation. Among these, Pn and Tr can serve as sensitive physiological indicators for evaluating metal toxicity.

#### 3.1.2 Exposure to HMs activates antioxidant defense

The antioxidant enzyme system is the first line of defense for plants to scavenge ROS. After HM exposure, although the overall level of the antioxidant enzyme group showed an increasing trend (4.31%), it did not reach statistical significance (*P* > 0.05, *n* = 198, **Figure 2**). This result indicates that the direction and magnitude of responses to metal stress vary considerably among different antioxidant enzymes, leading to a non-significant overall effect. Among the changes in specific enzymes, POD and CAT activities were significantly upregulated by 11.59% and 11.63%, respectively (*P* < 0.05), indicating that the H_2_O_2_ scavenging system is activated under metal stress. Although SOD activity was upregulated by 9.75%, it did not reach a significant level (*P* > 0.05). Notably, both APX and GR activities showed decreasing trends, with APX decreasing by 21.33% (*P* > 0.05), while GR decreased very significantly by 35.35% (*P* < 0.001, **Figure 2**).

The significant downregulation of GR is a notable finding within the antioxidant enzyme group. GR is a key enzyme in the ascorbate-glutathione cycle, responsible for reducing oxidized glutathione (GSSG) to GSH, thereby maintaining intracellular redox balance (Isik and Soydan, 2023; Kumar et al., 2020). The significant decrease in GR activity suggests a possible impairment of GSH regeneration capacity, which could be associated with a weakened cellular antioxidant defense (Lyubenova et al., 2007). This result is consistent with previous studies, such as the observed decrease in GR activity in *Panax notoginseng* under Cd stress (Ali et al., 2006). Furthermore, although APX did not decrease significantly, its negative trend further suggests that the ascorbate-glutathione cycle may be inhibited (Kaur et al., 2011; Vuletic et al., 2014). Therefore, despite the significant activation of POD and CAT under HM exposure, the very significant downregulation of GR may represent a key limiting link in the antioxidant defense system of *Panax* plants. This possibility could be verified in the future through exogenous application of GSH or overexpression of the GR gene.

#### 3.1.3 Gene expression disorders caused by exposure to HMs

The regulation of gene expression levels is an early response of plants to HM stress. As shown in **Figure 2**, HM exposure significantly upregulated the overall level of the gene expression group in *Panax* medicinal plants (56.10%, *P* < 0.01, *n* = 54), and the response magnitudes varied greatly among different genes. PgCAT and P450 transcription were very significantly upregulated by 161.22% (*P* < 0.001) and 167.92% (*P* < 0.001), respectively, making them the two indicators with the strongest responses in the gene expression group. PgAPX was also significantly upregulated by 80.04% (*P* < 0.001). These results are closely related to the significant upregulation of CAT at the enzyme activity level (11.63%), indicating that CAT is activated at both the transcriptional and enzymatic levels. The very significant upregulation of P450 transcription is particularly noteworthy (Fang et al., 2026; Huang et al., 2014; Cao et al., 2017), as it participates in the synthesis of various secondary metabolites and the detoxification of xenobiotics. Its high expression under metal stress may be associated with the metabolism of medicinal components unique to *Panax* plants.

However, SOD1 and SOD2 were significantly downregulated by 69.13% (*P* < 0.05) and 32.08% (*P* < 0.001), respectively. This opposite trend suggests the possibility of a negative regulatory relationship between H_2_O_2_ accumulation and SOD gene transcription. SOD catalyzes the conversion of superoxide anion (O_2_^−^) to H_2_O_2_, and one hypothesis is that excessive H_2_O_2_ accumulation may feedback to suppress further SOD transcription, thereby preventing more severe oxidative damage; however, this interpretation remains speculative and requires direct experimental validation. Although PgGPX showed a 3.85% upregulation, it did not reach a significant level (*P* > 0.05, **Figure 2**), suggesting that glutathione peroxidase may not be the primary peroxide-scavenging enzyme in *Panax* plants responding to metal stress. Overall, the gene expression group exhibited a significant upregulation (56.10%, *P* < 0.01), indicating that transcriptional regulation is an important component of the antioxidant defense response in *Panax* plants (**Figure 2**). Among the responsive genes, PgCAT and P450 showed the largest upregulation, suggesting they may represent key transcriptional events involved in the response to HM stress; however, this hypothesis requires further functional validation.

#### 3.1.4 Effects of HM exposure on stress and metabolism

The stress and metabolism group contained multiple key indicators reflecting the degree of oxidative damage, metabolite levels, and metal enrichment capacity. As shown in **Figure 2**, the overall response magnitude of this group was as high as 137.33% (*P* < 0.001, *n* = 254), making it the most responsive category among all four groups, indicating that HM exposure triggers severe oxidative stress and metabolic disorders in *Panax* plants (Geng et al., 2023; Wang et al., 2025b). Among the specific indicators, HM accumulation increased by 218.55% (*P* < 0.001, *n* = 174), demonstrating that *Panax* medicinal plants have a strong capacity for metal enrichment, which is both the reason for their susceptibility to metal toxicity and a reflection of their potential application value as soil remediation plants. Excessive accumulation of metals in plant tissues directly induces a burst of ROS, subsequently leading to oxidative damage (Kim et al., 2020; Zhang et al., 2025a; Castro-Aceituno et al., 2016). MDA and H_2_O_2_ increased significantly by 40.73% (*P* < 0.001) and 39.22% (*P* < 0.001), respectively, confirming the occurrence of lipid peroxidation and ROS burst.

Notably, although GSH increased by 14.97%, it did not reach statistical significance (*P* > 0.05, *n* = 13). This result is related to the very significant downregulation of GR activity (−35.35%); the inhibition of GR activity leads to a decreased capacity for GSH regeneration, so despite the possible induction of GSH synthesis, its net accumulation was not significant. ATP content decreased by 7.71% (*P* > 0.05). Although this decrease was not significant, the downward trend suggests that metal stress may exert a certain inhibitory effect on energy metabolism, which is consistent with the observed significant decrease in photosynthesis (Pn: −28.74%). The strong response of the stress and metabolism group (137.33%) clearly reveals the overall status of *Panax* medicinal plants after HM exposure. HMs, through substantial accumulation, are associated with a ROS burst, lipid peroxidation damage, and disruption of the antioxidant cycle, which may collectively contribute to the inhibition of energy metabolism. Although some antioxidant enzymes (POD, CAT) and genes (PgCAT, PgAPX) are activated, overall, they are still unable to completely prevent the occurrence of oxidative damage.

### 3.2 Predictive modeling and factor contribution analysis

**Figure 3:**
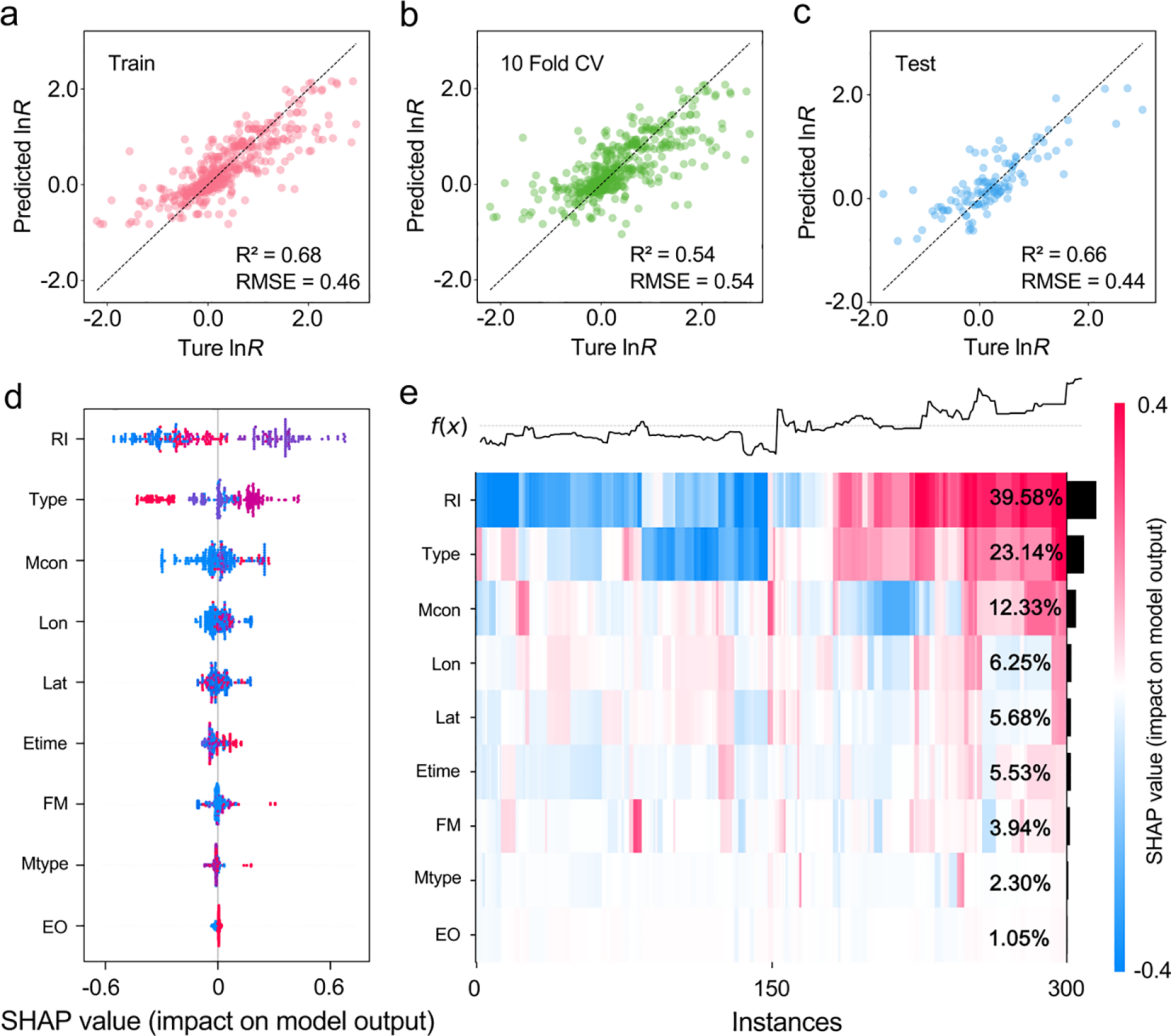
Predictive performance and feature importance analysis of the machine learning model. Comparison of the predictive performance of the RF model in the training set **(A)**, ten-fold cross-validation **(B)**, and test set **(C)**. **(D, E)** Ranking of feature importance contributions of predictors in the RF model. RI, response indicators; Type, category group (i.e. Physiological stress, Antioxidant enzyme, Gene expression, Stress and Metabolism); Mcon, concentration of HMs; Lon, longitude; Lat, latitude; Etime, exposure time; FM, field management; Mtype, HM types; EO, exposed object.

To identify the key driving factors affecting the toxic effects of HMs on *Panax* medicinal plants and to construct a predictive response model, this study employed the RF algorithm to build a toxicity effect prediction model. In terms of model prediction performance, the RF model demonstrated good fitting ability in the training set, with a R^2^ of 0.68 and a RMSE of 0.46 (**Figure 3A**). This indicates that the model can explain approximately 68% of the variation in effect sizes, and the selected features have good predictive capability for the antioxidant defense response of *Panax* plants under HM stress. To evaluate the stability and generalization ability of the model, we further performed ten-fold cross-validation. The results showed that the ten-fold cross-validation achieved an R^2^ of 0.54 and an RMSE of 0.54 (**Figure 3B**). The cross-validation performance was slightly lower than that of the training set but remained within an acceptable range, indicating that the model did not exhibit significant overfitting and had good robustness. On the independent test set, the model achieved an R^2^ of 0.66 and an RMSE of 0.44 (**Figure 3C**), with performance comparable to that of the training set, further validating the generalization ability of the RF model to new data. In summary, the RF model constructed in this study can reliably predict the magnitude of toxic effects on *Panax* medicinal plants under HM exposure, providing a credible foundation for subsequent feature importance analysis.

It is important to emphasize that the strong predictive performance of the full model (R^2^ = 0.66) is largely driven by the inclusion of response indicator and category group, which together explain the majority of variance in effect sizes. The low R^2^ (0.18) of the reduced model indicates that environmental variables alone cannot accurately predict the magnitude of toxicity responses across different endpoints. Consequently, the primary value of the RF analysis lies in ranking the relative importance of environmental drivers (as shown by SHAP) rather than in providing a general-purpose toxicity prediction tool. Any future application of the model for prediction should be restricted to the same set of endpoints and experimental conditions as those in the training data.

To explain the prediction mechanism of the model and quantify the contribution of each input feature to the toxic effect, we employed the SHAP method for feature importance analysis. As shown in **Figures 3D, E**, the contributions of the nine feature variables to model prediction differed significantly, with their percentage contributions ranked from highest to lowest as follows: response indicator (39.58%), category group (23.14%), metal concentration (12.33%), experimental site longitude (6.25%), experimental site latitude (5.68%), HM exposure duration (5.53%), field management practice (3.94%), heavy metal type (2.30%), and exposed object (1.05%). Among the environmental and experimental predictors, exposure duration ranked highest (28.61%), followed by metal concentration (26.13%) and geographical characteristics (latitude: 18.72%, longitude: 13.22%), with this ranking remaining stable in the reduced model. This indicates that these factors reliably modulate HM toxicity responses in *Panax* plants beyond the variations attributable to endpoint identity.

Response indicator and category group were the most important features influencing model prediction, together contributing over 60% (62.72%) of the predictive power. This finding is consistent with the established understanding, namely that different toxicity endpoints exhibit vastly different response magnitudes to HM exposure (e.g., metal accumulation at 218.55% vs. SOD1 at −69.13%), so is the primary factor associated with the effect size. Meanwhile, the high contribution of category group (23.14%) indicates that classifying the 22 indicators into four major categories—physiological stress, antioxidant enzyme, gene expression, and stress and metabolism—is biologically reasonable, as the response direction and intensity differ significantly among categories. Secondly, metal concentration was the most important feature among environmental and experimental factors (12.33%), with its importance significantly higher than that of heavy metal type (2.30%) and exposure duration (5.53%). This result aligns with the basic principle of dose-response relationships, namely that within a certain concentration range, higher HM concentrations lead to more severe oxidative stress and physiological damage in plants (Garcia et al., 2025; Georgiadou et al., 2018; Kuang et al., 2025). In contrast, the contribution of exposure duration was relatively low (5.53%), which may be related to the wide range of exposure durations included in this study and the variability across different studies. The combined contribution of experimental site longitude and latitude was approximately 11.93%, indicating that geographical representativeness (e.g., regional pollution background, climatic conditions) also has a certain impact on the metal stress response of *Panax* plants (Li et al., 2022; Tang et al., 2022). The contributions of field management practice (3.94%) and exposed object (1.05%) were relatively small, suggesting that under these study conditions, the effects of plant species (exposed object) and anthropogenic management practices on toxicity are weaker than those of metal concentration and indicator type themselves. Notably, the contribution of heavy metal type in the SHAP analysis was only 2.30%, much lower than that of metal concentration (12.33%). This result may indicate that, in *Panax* medicinal plants, the antioxidant defense responses induced by different HMs (e.g., Cd, Pb, As) share certain similarities, and the severity of stress depends more on the exposure concentration of the metal than on the metal species.

### 3.3 Sensitivity factors and pathway analysis

**Figure 4:**
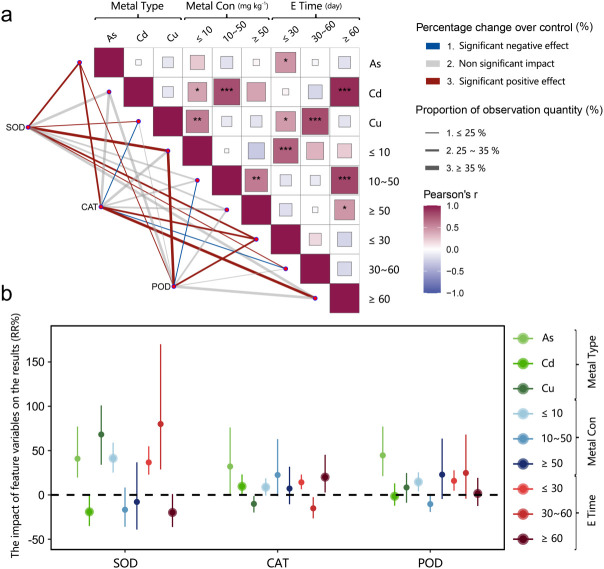
Effects of sensitive factors on the activities of common antioxidant enzymes. Pearson correlation analysis and influence trends among different levels of environmental variables **(A)**. Effects of different levels of environmental variables on the activities of common antioxidant enzymes **(B)**. Line colors indicate positive or negative effects of sensitive factors on antioxidant enzyme activities. Line thickness corresponds to the proportion of the 641 observational studies. Significance codes: ***P< 0.001, **P< 0.01, *,P< 0.05. SOD, superoxide dismutase; POD, peroxidase; CAT, catalase; E Time, exposure time.

**Figure 5:**
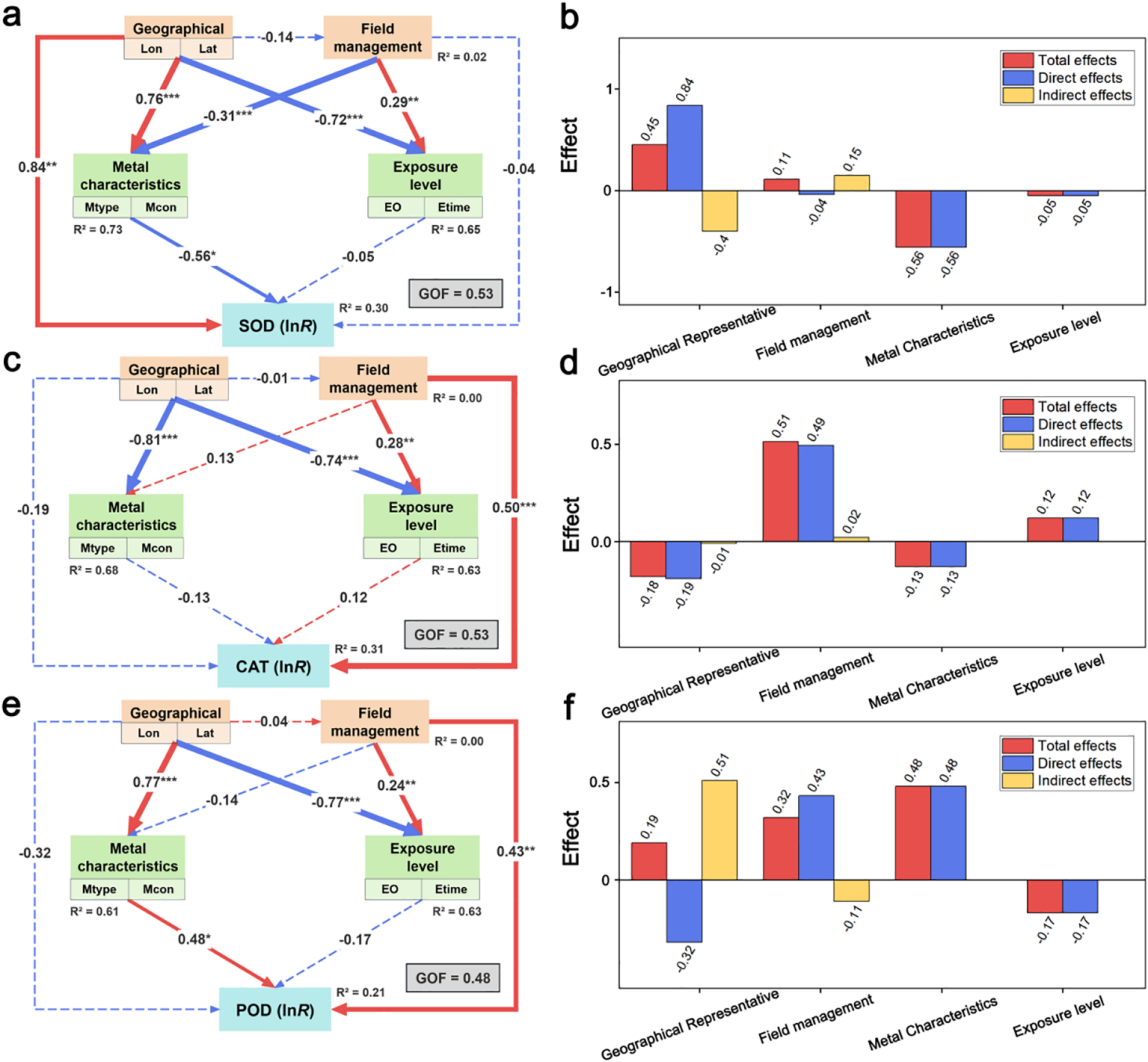
Path analysis of environmental drivers affecting common antioxidant enzyme activities. Partial least squares path modeling (PLS-PM) was applied to analyze the effects of environmental factors on SOD **(A, B)**, CAT **(C, D)**, and POD **(E, F)**. Colors indicate effect direction (red = positive, blue = negative), and path widths are scaled according to coefficient magnitudes (*P < 0.05, **P < 0.01, ***P < 0.001). ln*R*, logarithmic response ratio (effect value); SOD, superoxide dismutase; CTA, catalase; POD, peroxidase.

To systematically dissect the intrinsic relationships among HM type, concentration, exposure duration, and other factors, as well as their direct and indirect pathways affecting the antioxidant enzyme system, this study integrated correlation analysis (**Figure 4A**), subgroup meta-analysis (**Figure 4B**), and PLS-PM (**Figure 5**) to identify sensitive factors and clarify their multi-layered regulatory network. The results showed that different HM types exhibit differential regulation of antioxidant enzyme activities. For example, As was significantly positively correlated only with short-term exposure (*P* < 0.05), while Cu was positively correlated with low concentration and short-term exposure (*P* < 0.01) (**Figure 4A**). In the subgroup analysis, As very significantly activated SOD (40.87%), CAT (32.06%), and POD (44.64%) (*P* < 0.001 to 0.05); Cu very significantly activated SOD (68.19%) but significantly inhibited CAT (−10.10%) (*P* < 0.05); Cd had no significant direct effect on any of the three enzymes (**Figure 4B**). In the PLS-PM, metal characteristics showed a significant negative effect on SOD (path coefficient: *β* = −0.56, *P* < 0.01), but a positive effect on POD (*β* = 0.48, *P* < 0.05) (**Figure 5**). As, as a strong oxidizing metalloid, has been reported to be associated with ROS bursts and broad activation of the antioxidant enzyme system, suggesting it may be a potent stressor among the three metals (Nowicka, 2022; Tuncsoy and Tuncsoy, 2023); Cu may generate specific oxidative signals by interfering with the chloroplast electron transport chain, which could correlate with SOD upregulation and CAT inhibition (Ahmed et al., 2010; Aziz et al., 2020; Toteja et al., 2017); although Cd is often associated with high-concentration long-term exposure, its toxicity is more often manifested as indirect metabolic interference (e.g., displacing essential ions or inducing metal accumulation) rather than direct regulation of enzyme activity (Zhou et al., 2020; Alfadul and Al-Fredan, 2013; Gharib and Ahmed, 2023). Therefore, risk assessment should distinguish the key effects of different HMs, such as focusing on acute enzyme induction for As and emphasizing cumulative exposure dose for Cd.

The combined effect of metal concentration and exposure duration regulated the enzymatic response in a stage-dependent manner. Subgroup analysis showed that low concentration (≤10 mg kg^-1^) and short-term exposure (≤30 days) both significantly activated SOD (41.27% and 36.70%) and POD (14.64% and 15.88%) activities (*P* < 0.001 to 0.01), whereas no significant changes were observed for either enzyme under medium-to-high concentration or long-term exposure. The pattern of SOD and POD being induced by low concentration and short-term exposure, while showing no significant response to high concentration and long-term exposure, suggests a possible threshold process in plant antioxidant defense (González-Reguero et al., 2024; Bourgeault et al., 2013). Under mild stress, the enzyme system is rapidly mobilized to scavenge reactive oxygen species, but when the tolerance limit is exceeded or stress persists, enzyme synthesis is inhibited or inactivation occurs (Aihemaiti et al., 2019). In contrast, CAT exhibited a cyclic response process of short-term activation (14.22%), medium-term inhibition (−15.10%), and long-term reactivation (20.05%) (*P* < 0.001 to 0.05). This may stem from compensatory mechanisms at different stages: short-term response to elevated H_2_O_2_, medium-term activity decline due to protein oxidative damage, and long-term adaptive upregulation through transcriptional reprogramming (Mora et al., 2022; Shiry et al., 2023). Exposure level was significantly negatively regulated by geographical characteristics in all three antioxidant enzyme path models (*β* = −0.72 to −0.77, *P* < 0.001), but the direct path from exposure level to enzyme activity did not reach significance, indicating that the effect of exposure duration is largely achieved through interactions with other factors (e.g., metal concentration). Therefore, sampling at a single time point may underestimate the true CAT response, and future studies should focus on temporal dynamics. Meanwhile, the model explained a modest portion of variance in enzyme activities (SOD: R^2^ = 0.30; CAT: R^2^ = 0.31; POD: R^2^ = 0.21), suggesting that unmodeled factors contribute substantially to antioxidant responses.

Geographical characteristics and field management have the potential to intervene in the toxic effects of HM exposure on *Panax* medicinal plants. PLS-PM clearly showed that geographical characteristics were the most upstream driving factor in all three path models. In the SOD model, geographical characteristics had a significant direct positive effect on SOD activity (*β* = 0.84, *P* < 0.01), while also exerting indirect effects by promoting metal characteristics (*β* = 0.76) and suppressing exposure level (*β* = −0.72). In the CAT and POD models, geographical characteristics showed a very significant negative effect on exposure level (CAT: *β* = −0.74, POD: *β* = −0.77), but the direction of their effects on metal characteristics was opposite (CAT: *β* = −0.81, POD: *β* = 0.77). Geographical characteristics not only represent regional pollution backgrounds (e.g., mining areas, industrial areas, and agricultural areas) but also imply factors such as climate and soil type (Ren et al., 2019; Yang et al., 2022; Nowbakht et al., 2025), which may collectively influence the bioavailability of HMs and the basal antioxidant capacity of plants. The inconsistency in the direction of regulation by geographical characteristics on different enzymes may reflect the adaptive divergence of CAT and POD in evolution to different ecological and geographical environments. Field management acted as a positive regulatory factor in all three models, with particularly significant direct effects on CAT (*β* = 0.50, *P* < 0.001) and POD (*β* = 0.43, *P* < 0.01), while also having a positive effect on exposure level (*β* = 0.24 to 0.29, *P* < 0.01). This suggests that field management, as an artificially intervenable factor, may have a positive effect, particularly on CAT and POD activities. This may provide feasible agronomic pathways for practical production, such as through the application of organic fertilizer, regulation of soil pH, or rational irrigation, which can directly enhance the antioxidant enzyme activities of *Panax* medicinal plants, thereby alleviating damage from metal stress.

### 3.4 Limitations

Although this study systematically analyzed the response characteristics of the antioxidant defense system of *Panax* medicinal plants under HM stress, several limitations remain. First, our meta-analysis included multiple effect sizes extracted from the same studies, which may introduce hierarchical dependence and potential pseudoreplication. Our sensitivity analysis (1,000 iterations, one effect size per study) confirmed robustness (sign consistency > 98%, median deviation< 15%). Nonetheless, future studies should employ multilevel meta-analysis or robust variance estimation to more rigorously account for within-study correlations. Second, the overrepresentation of pot experiments (over 80%) suggests that the geographic signal captured by longitude and latitude may primarily reflect soil-source regional differences rather than *in-situ* field conditions, and extrapolation of these findings to field scenarios should be made with caution. Finally, the field management variable exhibited substantial class imbalance, with the majority of observations belonging to the none/control category. This imbalance may limit the statistical power to differentiate the effects of specific amelioration practices and should be addressed in future studies through more balanced experimental designs or targeted data collection. The PLS-PM latent variables geographical characteristics and field management are broad composites; future studies should incorporate directly measured soil and climate variables. Furthermore, the extremely high heterogeneity (I^2^ = 99.84%) warrants additional caution when interpreting the overall pooled effect sizes. This level of variability indicates that the included studies differ substantially across multiple dimensions, including toxicity endpoint categories, Panax species, heavy metal types and concentrations, exposure durations, and experimental systems (pot vs. field). Consequently, the global summary estimates (e.g., the overall 4.31% change in antioxidant enzyme activities) should be viewed as average tendencies rather than universal conclusions. The true value of our meta-analysis lies not in these overall averages but in the stratified analyses, the identification of key modulating factors via machine learning, and the pathway elucidation via PLS-PM. Readers are encouraged to interpret endpoint-specific and subgroup-specific effect sizes (**Figure 2**) as the primary findings.

## 4 Conclusions

This study, by combining meta-analysis with machine learning modeling, characterized the response patterns and potential regulatory mechanisms of the antioxidant defense network in *Panax* medicinal plants under HM stress. The results showed that HM exposure significantly inhibited photosynthetic physiology (net photosynthetic rate and transpiration rate decreased by approximately 30%), caused substantial metal accumulation (218.55%), and induced oxidative damage (malondialdehyde and hydrogen peroxide increased by approximately 40%). The very significant downregulation of glutathione reductase (−35.35%) suggests that it may be a key weak link in the antioxidant defense system. The full RF model (including endpoint categories) showed good fit (R^2^ = 0.66, RMSE = 0.44), and model-based SHAP analysis indicated that metal concentration (12.33%) is a more important predictor than metal type (2.30%). PLS-PM analysis revealed that appropriate field management strategies are positively associated with antioxidant enzyme activities under stress conditions, suggesting a potential role in alleviating physiological damage caused by HM stress. In summary, the systematic analytical framework established in this study clarifies the action pathways and key regulatory factors of HM stress, providing theoretical support for risk management of HM contamination and stress-resistant cultivation of *Panax* medicinal plants.

## Data Availability

The original contributions presented in the study are included in the article/**Supplementary Material**. Further inquiries can be directed to the corresponding author/s.
