# Supplementary material for: Antioxidant defense network and toxicity risk assessment in Panax species under heavy metals stress
Source: Front Plant Sci. 2026 Jun 15;17:1860085. doi: 10.3389/fpls.2026.1860085 (PMC13310740; doi:10.3389/fpls.2026.1860085)
Supplement: Supplementary file 1 [file DataSheet1.docx]

Supplementary Material


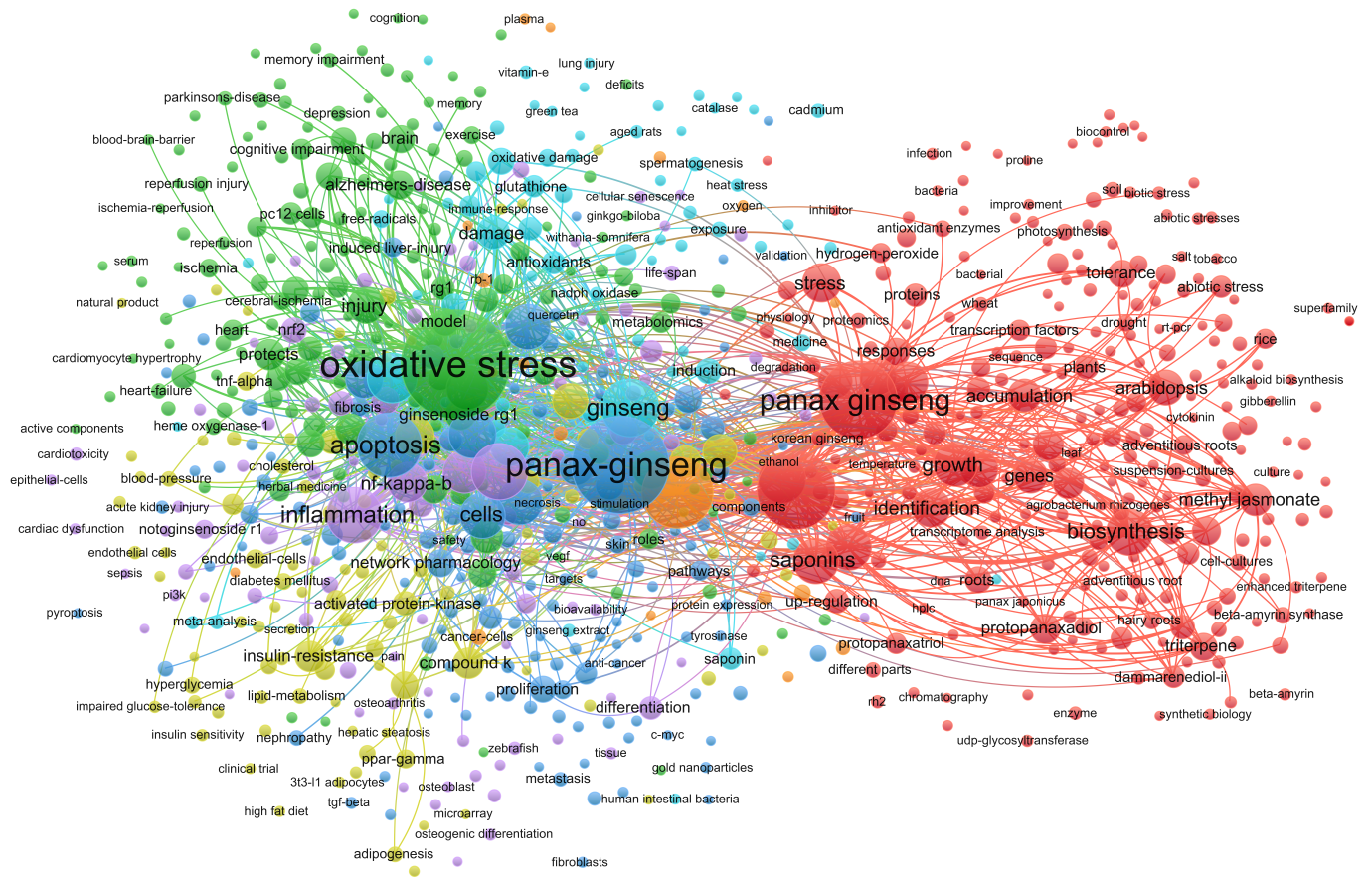


**Supplementary Figure 1.** A keyword contribution network diagram drawn using VOS viewer software. The node size represents the frequency of occurrence (≥ 5).

**
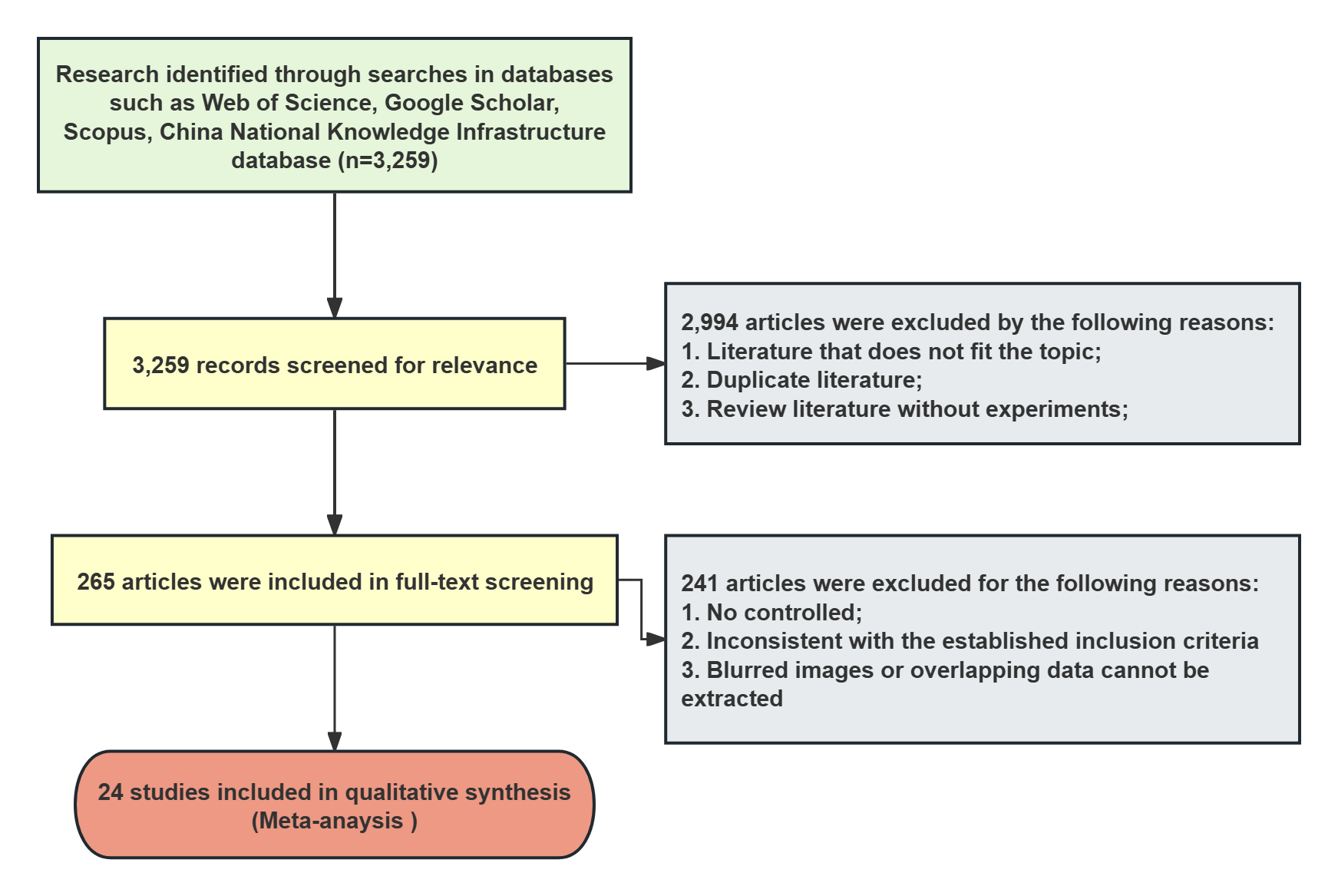
**

**Supplementary Figure 2.** In the meta-analysis, a schematic diagram of the retrieval and screening process for 24 included literature is provided.

**
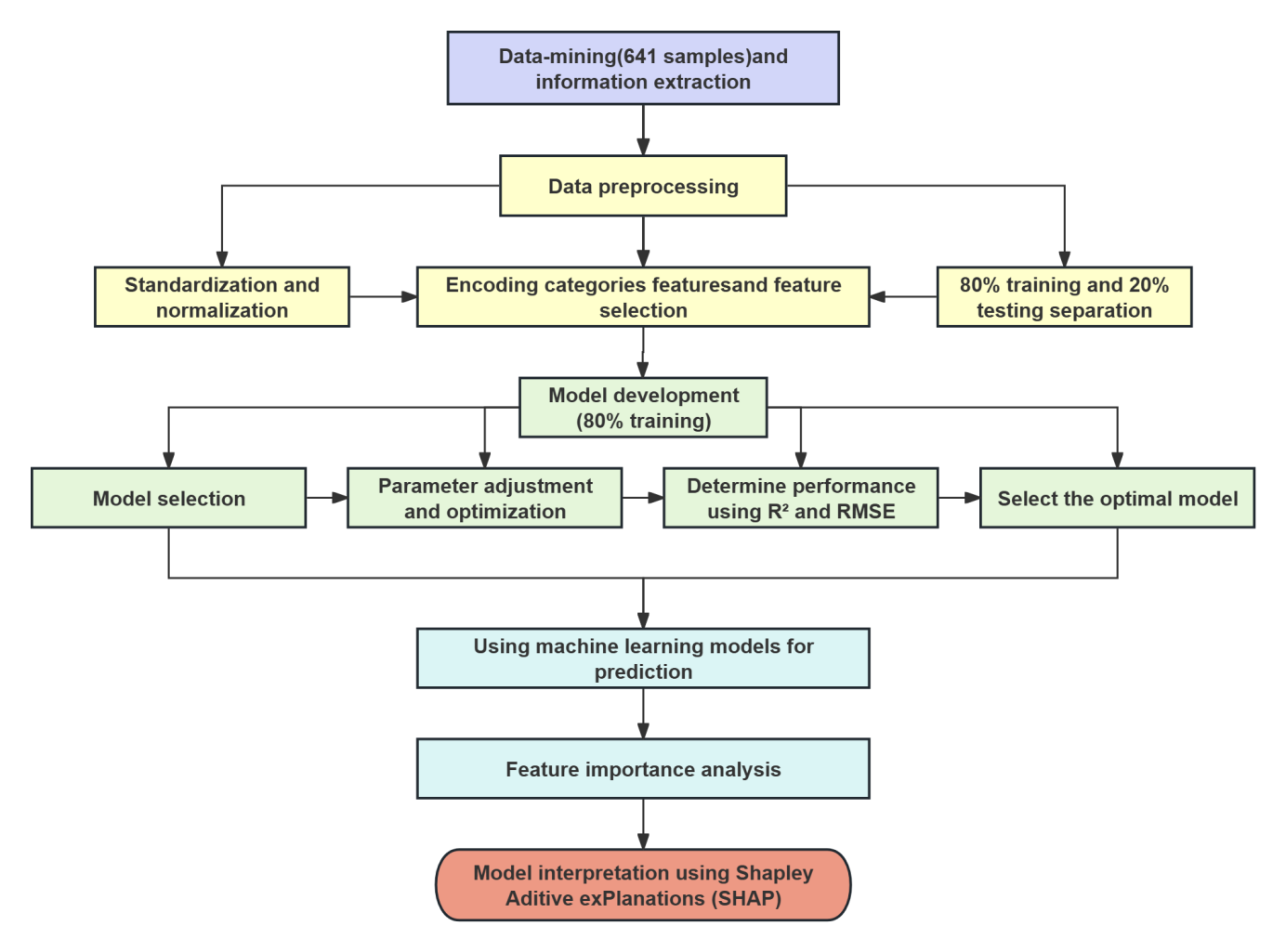
**

**Supplementary Figure 3.** The construction process of machine learning models based on SHAP value interpretability.
